# Myocarditis Secondary to Human Monocytotrophic Ehrlichiosis

**DOI:** 10.7759/cureus.59369

**Published:** 2024-04-30

**Authors:** Vidhu Kariyawasam, Kairav Shah

**Affiliations:** 1 Infectious Diseases, University of Florida, Gainesville, USA; 2 Infectious Diseases, Southern Regional Medical Center, Atlanta, USA

**Keywords:** tick-borne illness, zoonotic infections, anaplasmosis, ehrlichiosis, myocarditis

## Abstract

Ehrlichiosis is a tick-borne illness that has been recognized as a source of human infection with increased incidence in the United States over the last decade. The usual presentation is with acute febrile illness, myalgia, malaise with confusion, and central nervous system abnormalities, along with laboratory data concerning transaminitis and hematological abnormalities. Though many complications have been associated with Ehrlichiosis, very few cardiac complications have been reported. We report a rare presentation of Ehrlichiosis in a 63-year-old female who presented with acute fever, transaminitis, and renal failure followed by the development of myocarditis. As part of the diagnostic work-up, an examination of the peripheral smear revealed intracytoplasmic granules in monocytes, which were later confirmed through serology to have Ehrlichia chaffeensis (E. chaffeensis). Given the high degree of initial clinical suspicion, the patient was started on empiric doxycycline and fully recovered with no disease-associated sequelae.

## Introduction

Ehrlichiosis is a term used to describe infections of organisms of Anaplasmataceae of the order of Rickettsiales [1]. They are obligate intracellular Gram-negative bacteria that grow within membrane-bound vacuoles in human and animal leukocytes. There at least five species that have been identified to cause disease in humans, and among them are Ehrlichia chaffeensis (E. chaffeensis), which causes human monocytic ehrlichiosis (HME), and Anaplasma phagocytophilum which causes human granulocytic anaplasmosis (HGA) [2], are the commonest. Usually, E. chaffeensis presents as an acute febrile illness associated with neurological, hematological, and liver abnormalities [3]. Myocarditis remains an uncommon presentation of Ehrlichiosis.

## Case presentation

A 63 year-old-female with hypothyroidism, carpal tunnel syndrome, and seasonal allergies presented to an urgent care clinic with fevers, dysuria, right-sided flank pain, and abdominal pain. She was given trimethoprim-sulfamethoxazole (TMP-SMX) for a presumed urinary tract infection. However, with no improvement after three days of antibiotic use and worsening fevers along with nausea and vomiting, she presented to the hospital. Three weeks prior, she had returned from a nine-month stay in Tajikistan, Central Asia. She denied any mosquito or tick bites. Her home in Central Florida was in a wooded area. She was a retired nurse and denied high-risk sexual behavior.

On physical exam, she was hypotensive and febrile to 39^o^Celsius. Other vital signs were normal. She appeared lethargic and drowsy, and an abdominal exam was pertinent for tenderness in the right upper quadrant without peritoneal signs. She had no meningeal signs and had a normal cardiopulmonary examination.

On laboratory evaluation, the complete blood count showed mild leukopenia, mild anemia, and thrombocytopenia; the chemistry panel showed hyponatremia, acute kidney injury, and transaminitis. She was also noted to have indirect hyperbilirubinemia and low haptoglobin. Initial urine analysis showed no pyuria, and urine culture was negative. Blood cultures were also negative.

Chest X-ray showed mild basilar atelectasis and small left-sided pleural effusion. Computed tomography (CT) of the abdomen and pelvis without contrast did not identify acute abdominal pathology or urolithiasis. Ultrasound (US) of the abdomen showed splenomegaly measuring up to 15.6 cm and cholelithiasis without acute cholecystitis. The patient was admitted for further work-up of fevers and lab abnormalities.

Three days after admission, she developed typical chest pain with a pressure-like sensation in the chest. Cardiac enzymes were elevated with troponin I at 2.37 (normal: <0.04 ng/mL) and creatine kinase-myoglobin binding (CK-MB) at 9.6 ηg/mL). The electrocardiogram showed low voltage in all leads and T wave flattening in inferior leads. Echocardiography of the heart showed a normal ejection fraction of 60%, mildly dilated left atrium, and mild to moderate mitral regurgitation. She underwent left heart catheterization, which showed normal coronary arteries. She subsequently underwent a cardiac MRI to rule out myocarditis. The cardiac MRI done in the outpatient setting after discharge was suggestive but not conclusive for myocarditis, given suboptimal lead gadolinium enhancement. Given that the patient had chest pain with elevated troponin with cardiac catheterization showing no blockage in the coronary arteries and subsequent Cardiac MRI findings along with the complete resolution of symptoms with doxycycline alone, a diagnosis of Ehrlichia-induced myocarditis was made. Epstein-Barr virus (EBV Viral Capsid antigen IgM

**Figure 1:**
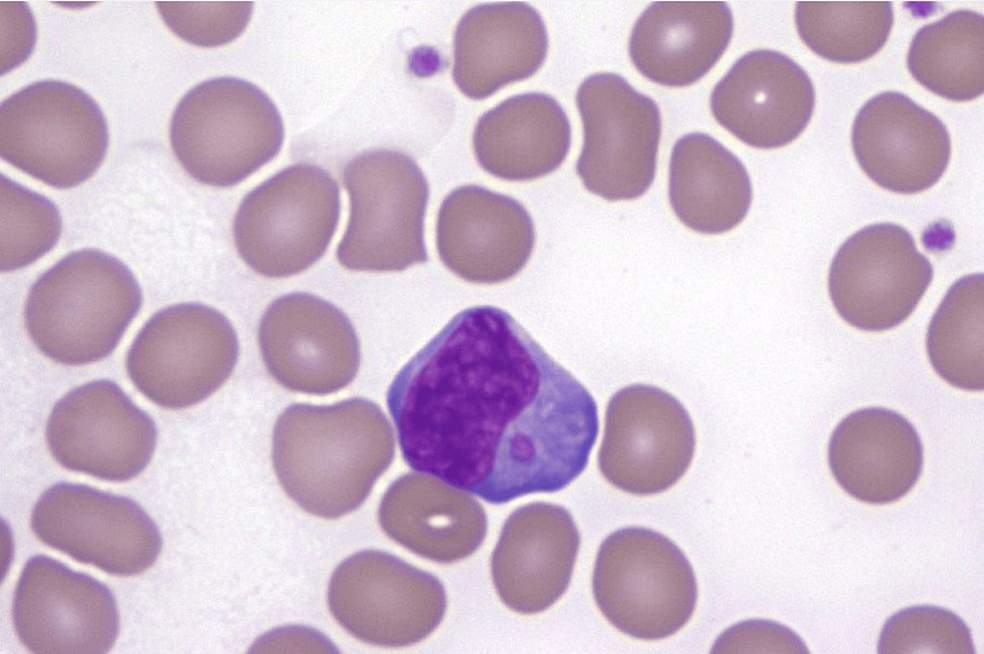
Intracytoplasmic eosinophilic morula in monocyte on peripheral smear. Hematoxylin and eosin stain, magnification x500.

With multi-organ involvement and travel history, malaria was ruled out by negative malaria antigen (Binax ®) and negative thick and thin peripheral smears. Acute cytomegalovirus (CMV) and Epstein-Barr virus (EBV) infections were also ruled out with negative EBV viral capsid antigen (VCA) IgM and undetectable EBV and CMV DNA PCR. Lyme disease antibody, Rocky Mountain spotted fever (RMSF) IgG and IgM, Brucella antibody, and Leishmania IgG were all negative. Acute hepatitis panel (including hepatitis A, B, and C) was negative. A peripheral smear of blood showed intracytoplasmic granules in monocytes, leading to high suspicion of diagnosis of Ehrlichiosis (Figure 1). Ehrlichia antibody panel was also initially negative but turned positive on hospital day nine.

Given the pattern of laboratory involvement, including hematological abnormalities (leukopenia, thrombocytopenia) and evidence of end-organ injury (transaminitis and renal failure), despite the absence of a history of tick bite, empiric doxycycline was initiated on hospital day one which was continued to complete a three-week course and resulted in resolution of her symptoms. Post-hospital follow-up at four weeks in the clinic showed complete resolution of transaminitis, creatinine, and hematologic abnormalities.

## Discussion

Ehrlichiosis was initially thought to be a zoonotic rickettsial disease. The first diagnosed case of human Ehrlichiosis in the United States occurred in a 51-year-old male from Arkansas in 1986, with the main causative agent found to be E. chaffeensis [4]. Since then, there has been an increasing incidence of Ehrlichiosis in the United States, particularly in the southcentral and southeastern United States. The average reported annual incidence of HME in the United States was 3.2 cases/per million population in 2012, with wide variation in incidence by state [5]. The main vector of transmission is thought to be Amblyomma americanum, commonly known as the lone star tick.

Usually, E. chaffeensis presents as an acute febrile illness with associated central nervous system (CNS), hematological, and liver abnormalities. However, it can have a wide spectrum of presentations, even in the immunocompetent individual, from a short, self-limiting illness with non-specific symptoms to severe multisystem illness. In documented cases of Ehrlichiosis, 49% require hospitalization and is life-threatening in at least 9.2% [6].

Though multiple severe complications, including acute respiratory distress syndrome, disseminated intravascular coagulation, acute renal and liver failure, and CNS abnormalities, have been reported, there have been very few reports of cardiac complications with Ehrlichiosis. Myocarditis remains an uncommon complication of human Ehrlichiosis. The true pathogenesis leading to myocarditis remains unclear. Immunohistochemical staining demonstrated the organism in the cytoplasm of inflammatory cells in autopsy specimens of perivascular myocardial tissue. However, it is uncertain whether this directly induces myocardial damage [7] or if it induces transient immunosuppression and concomitant inflammatory cell dysfunction that causes non-specific damage to myocytes. This remains a rare finding, with only three documented cases of Ehrlichiosis (one resulting in fatality) causing myocarditis, with HGA more common than HME [8-10].

Seroconversion, as in our patient, can be delayed (initial IgG was negative on day one and day four and only positive on day nine) and can delay early diagnosis. Therefore, careful examination of peripheral smears and relaying clinical suspicion to the pathologist can lead to identifying intracytoplasmic morulae. A thorough peripheral smear examination can identify 20-75% of infections [11]. Timely diagnosis and treatment have been shown to reduce morbidity and mortality [12]. Once symptoms have continued beyond one week, optimal therapy becomes progressively less effective.

## Conclusions

In conclusion, Ehrlichiosis can present as an acute febrile illness with non-specific findings and can lead to multi-organ dysfunction, with myocarditis being an uncommon presentation. Serological diagnosis can be delayed, and in a patient with high clinical suspicion, a close examination of the peripheral smear can guide early and appropriate antimicrobial therapy with doxycycline. It can prevent cardiac complications, long-term sequelae, and fatalities and lead to complete recovery.
